# The burden of dermatitis from 1990–2019 in the Middle East and North Africa region

**DOI:** 10.1186/s12889-024-17836-z

**Published:** 2024-02-07

**Authors:** Saeid Safiri, Mehran Jaberinezhad, Seyed Ehsan Mousavi, Kimia Motlagh Asghari, Ali Shamekh, Seyed Aria Nejadghaderi, Mark J. M. Sullman, Yousef Houshyar, Elham Behrangi, Ali-Asghar Kolahi

**Affiliations:** 1 Social Determinants of Health Research Center, Department of Community Medicine, School of Medicine, Tabriz University of Medical Sciences, Tabriz, Iran; 2 Clinical Research Development Unit of Tabriz Valiasr Hospital, Tabriz University of Medical Sciences, Tabriz, Iran; 3 Neurosciences Research Center, Aging Research Institute, Tabriz University of Medical Sciences, Tabriz, Iran; 4 Physical Medicine and Rehabilitation Research Center, Aging Research Institute, Tabriz University of Medical Sciences, Tabriz, Iran; 5 Systematic Review and Meta-Analysis Expert Group (SRMEG), Universal Scientific Education and Research Network (USERN), Tehran, Iran; 6 Department of Life and Health Sciences, University of Nicosia, Nicosia, Cyprus; 7 Department of Social Sciences, University of Nicosia, Nicosia, Cyprus; 8 Department of Dermatology, Özel Çankaya Hospital, Barbaros, Ankara, Turkey; 9 Department of Dermatology, Rasool Akram Medical Complex Clinical Research Development Center (RCRDC), School of Medicine, Iran University of Medical Sciences, Tehran, Iran; 10 Social Determinants of Health Research Center, Shahid Beheshti University of Medical Sciences, Tehran, Iran

**Keywords:** Dermatitis, Middle East and North Africa, Epidemiology, Mortality, Year-lived with disability

## Abstract

**Background:**

There are several types of dermatitis, each capable of causing enduring changes that extend beyond physical discomfort. In severe cases, dermatitis can significantly affect mental health, social interactions, and the overall quality of life. This study reports the burden of dermatitis in the Middle East and North Africa (MENA) region from 1990 to 2019, according to sex, age category, and socio-demographic index (SDI).

**Methods:**

Publicly available data regarding the point prevalence, incidence, and years lived with disability (YLDs) were collected from the Global Burden of Disease 2019 study for both the MENA region and its constituent countries. The point prevalence, incidence, and YLDs of dermatitis were represented as counts and age-standardised rates with 95% uncertainty intervals (UIs).

**Results:**

In 2019, the age-standardised point prevalence of dermatitis was 2744.6 (2517.8–3003.1) per 100,000 population, which was 2.3% lower than in 1990. The YLD rate was 92.3 (55.6–143.4) per 100,000 population, which was 3.1% lower than in 1990. The largest point prevalence rates were observed among those aged 70–74, for both sexes. The 2019 MENA/Global DALY ratio was not above one in any age group for either sex. During the period 1990 to 2019, there was no clear correlation between the burden of dermatitis and the SDI level.

**Conclusion:**

The dermatitis burden in the MENA region remained relatively stable from 1990 to 2019. Future prevention efforts should focus on improving healthcare access, health education, and workplace safety regulations.

**Supplementary Information:**

The online version contains supplementary material available at 10.1186/s12889-024-17836-z.

## Introduction

Skin and subcutaneous diseases are widespread health challenges that exert a significant burden on both individuals and the healthcare systems across the world [1]. Dermatitis, which includes atopic dermatitis, contact dermatitis and seborrheic dermatitis [1], is one of the most common skin diseases. Dermatitis, also known as eczema, refers to inflammation of the skin, which can be caused by various different factors, such inherent skin defects, direct contact with irritants, or allergic reactions [2–6]. Dermatitis is characterized by symptoms such as redness, itching, and occasionally blistering [2–6]. Atopic dermatitis, which commonly manifests itself in childhood and frequently coexists with asthma and other allergies, is a common type of dermatitis [2, 3]. In contrast, contact dermatitis occurs when the skin comes into direct contact with an irritating substance or allergen, leading to skin damage or the activation of immune-triggered symptoms [4]. Seborrheic dermatitis predominantly affects oily body areas, most notably the scalp, but can also affect the face, ears, chest, and other regions of the body. This condition gives rise to scaly patches, inflammation, and persistent dandruff [5, 6].

Although each type of dermatitis exhibits distinct characteristics, they can all cause enduring changes that extend beyond physical discomfort, and in severe cases can profoundly affect mental health, social interactions, and the overall quality of life [7–9]. Physically, dermatitis presents with inflamed, itchy rashes that can cover large areas of the skin, potentially leading to secondary infections [2–6]. The discomfort, itching and pain associated with moderate to severe disease can significantly disrupt daily activities, including school, work, and social interactions [7]. Furthermore, individuals may experience sleep disturbance, social isolation, diminished self-confidence, anxiety, and depression [8–10]. Living with dermatitis may also necessitate lifestyle adjustments during flares, such as avoiding certain activities or clothing choices [7–9]. Moreover, the burden extends to the families and caregivers of the affected individuals [11].

The burden of dermatitis is a pressing global health issue, since the disease burden is higher than that caused by skin tumors [1]. In 2013, atopic, contact, and seborrheic dermatitis collectively contributed to a significant share (0.38%) of the worldwide burden of disease [11]. In 2017, atopic dermatitis ranked as the 15th most prevalent nonfatal disease globally, imposing a substantial disability burden [10]. In 2019, the worldwide incidence of dermatitis was approximated at around 418 million cases each year [12]. Furthermore, the economic costs associated with dermatitis are substantial, encompassing both the direct and indirect expenses [13, 14]. Healthcare costs for individuals with atopic dermatitis significantly increase with the severity of the disease, and indirect costs, such as loss of productivity, further contributes to the burden [13, 14]. Studies have shown that in European countries, the annual costs per patient range from €1572 to €6993, primarily driven by expenses related to hospitalizations, outpatient visits, and medications [14]. In the United States, the annual cost of atopic dermatitis alone has been reported to be higher than $5 billion [15].

Despite its prevalence, there is limited information on dermatitis in the Middle East and North Africa (MENA) region, making it difficult to develop effective prevention and management strategies. Prior studies have described the burden of dermatitis and its subtypes globally, but this study provided limited data on the MENA region [16]. Given the wide-ranging implications of dermatitis, understanding its burden is crucial information for the policymakers and health authorities of the 21 countries in MENA. The current research utilized information from the Global Burden of Disease (GBD) 2019 study to analyse the prevalence, incidence and years lived with disability (YLDs) due to dermatitis in the 21 MENA countries between 1990 and 2019, by sex, age category, and SDI.

## Methods

### Overview

The GBD project is an extensive epidemiological initiative, run by the Institute for Health Metrics and Evaluation (IHME), which was established to monitor and record the epidemiological burden of injuries and diseases across the globe. The latest version of GBD, GBD 2019, collected information about 369 diseases and injuries across 204 countries and territories over the period 1990 to 2019 [12]. The MENA region contains 21 countries, including: Afghanistan, Algeria, Bahrain, Egypt, Iran (Islamic Republic of), Iraq, Jordan, Kuwait, Lebanon, Libya, Morocco, Oman, Palestine, Qatar, Saudi Arabia, Sudan, the Syrian Arab Republic, Tunisia, Turkey, the United Arab Emirates and Yemen. The methodology used for GBD 2019, and the improvements made since 2017, have previously been reported [12, 17]. Interested readers can access the data through these links: https://vizhub.healthdata.org/gbd-compare/ and http://ghdx.healthdata.org/gbd-results-tool.

### Data sources and case definition

Dermatitis is an inflammatory skin condition that disrupts the epidermal barriers. Atopic dermatitis is a chronic cutaneous condition marked by localized or widespread skin inflammation, which leads to symptoms like itching, dryness, and increased cutaneous activity, and is often accompanied by elevated serum immunoglobulin E levels. Contact dermatitis is a localized skin inflammation that results from direct contact with allergens or irritants. This condition manifest itself in various clinical presentations, ranging from asymptomatic to itching, stinging or pain. Seborrheic dermatitis is a recurrent skin condition that affects areas with a high density of sebaceous glands, causing erythematous and scaly patches.

In the International Classification of Diseases version 10 (ICD10), all conditions coded as L20, L22-26, and L21 were classified as atopic dermatitis, contact dermatitis, and seborrheic dermatitis, respectively [12]. Dermatitis data were gathered from countries across the world using the scientific literature and claims data [12]. The IHME conducted a literature review in GBD 2016, which included studies that reported the incidence and prevalence of dermatitis, with supplementary research and data being added based upon the recommendations of a team of skin specialists. For further details regarding the data utilized in the GBD 2019 study, please refer to: https://ghdx.healthdata.org/gbd-2019/data-input-sources.

### Disease model

The study utilized DisMod-MR 2.1 to separately model the prevalence of atopic dermatitis, contact dermatitis, and seborrhoeic dermatitis by sex, age category, year, and country. As the accessible data primarily consisted of prevalence information, IHME incorporated additional expert priors to better enrich the analyses. To estimate the prevalence of atopic dermatitis, a prior value of zero was set for excess mortality, and a prior value of 0 to 0.2 was used for remission. The crosswalks within DisMod were updated for GBD 2019 using the Meta Regression – Bayesian, Regularized, Trimmed (MR-BRT) modeling tool. Administrative records, including information from sources like the Medical Expenditure Panel Survey (USA), Marketscan 2000 (USA), and non-physical exam-based data were standardized to improve their alignment with the general population.

In order to estimate the prevalence of contact dermatitis, a prior value of zero was set for excess mortality, a prior value of 0.1 to 4 was used for remission, and the incident cases were set to zero for children under 6 years old. The MR-BRT Crosswalk Adjustment Factors were utilized again, and data were adjusted with a recall period of 12 months. Information from the Medical Expenditure Panel Survey, Market Scan 2000 data, and non-exam-based data were also adjusted to align with the other data points that better represented the characteristics of the general population. Again, crosswalks within DisMod were created utilizing the MR-BRT modeling tool. To estimate the prevalence of seborrheic dermatitis, a prior value of zero was set for excess mortality. The prior value of remission ranges from 0.1 to 12, which corresponded to a duration of 1 month to 10 years. Incident cases were set to 0–4 years (0–0.1) and 60–100 years (0–0.01). Again, the crosswalks within DisMod were updated using the MR-BRT modeling tool.

### Severity and years lived with disability

Table S1 presents the lay descriptions and disability weights (DWs) for each sequelae and severity level of dermatitis. Atopic dermatitis was divided into mild (DW = 0.027), moderate (DW = 0.188), and severe atopic dermatitis (DW = 0.576) [12]. In contrast, contact dermatitis was divided into mild (DW = 0.027) and moderate contact dermatitis (DW = 0.188) [12]. Symptomatic seborrheic dermatitis had a DW of 0.027 [12].

### Data analysis

The study also reported 95% uncertainty intervals (UIs) for every estimate, which were estimated by running 1000 iterations for each estimate, placing these in numerical order and selecting the 25th and 975th values. The GBD standard population was also utilized to standardize all estimates. Smoothing Splines models [18] were employed to investigate the connection between dermatitis-related YLDs and the SDI. The SDI metric is a comprehensive measure that encompasses average income per capita, average years of education (≥ 15 years old), and the total fertility rate (for those under 25 years old), spans from 0, representing the lowest development level, to 1, indicating the highest development level. All figures were produced utilizing R (Version 3.5.2).

## Results

### The Middle East and North Africa region

In 2019, there were more than 16.6 million prevalent cases (95% UI 15.2 to 18.1 million) of dermatitis in MENA, with an age-standardised point prevalence of 2744.6 per 100,000 population (95% UI 2517.8 to 3003.1), which was 2.3% lower than in 1990 (95% UI -3.2 to -1.5) (Table 1 and Table S2). Furthermore, there were 29.0 million incident cases (95% UI 25.0 to 33.3 million), with an age-standardized rate of 4844.4 (95% UI 4160.3 to 5539.7) per 100,000, which was unchanged (95% UI -0.2 to 0.1) since 1990 (Table 1 and Table S3). There were 565.3 thousand (95% UI 339.6 to 883.3) YLDs in the MENA region with an age-standardised rate of 92.3 (95% UI 55.6 to 143.4) per 100,000. The age-standardised YLD rate decreased by 3.1% from 1990 to 2019 (95% UI -4.6 to -1.6) (Table 1 and Table S4).

**Table 1:** Prevalence, incidence and YLDs due to dermatitis in 2019 and the percentage change in the age-standardised rates during the period 1990–2019

	**Prevalence (95% UI)**			**Incidence (95% UI)**			**YLD (95% UI)**		
	**Counts (2019)**	**ASRs (2019)**	**Pcs in ASRs 1990–2019**	**Counts (2019)**	**ASRs (2019)**	**Pcs in ASRs 1990–2019**	**Counts (2019)**	**ASRs (2019)**	**Pcs in ASRs 1990–2019**
North Africa and Middle East	16,581,955 (15,229,446, 18,129,071)	2744.6 (2517.8, 3003.1)	-2.3 (-3.2, -1.5)	28,989,852 (24,931,092, 33,310,421)	4844.4 (4160.3, 5539.7)	0 (-0.2, 0.1)	565,253 (339,590, 883,269)	92.3 (55.6, 143.4)	-3.1 (-4.6, -1.6)
Afghanistan	1,006,275 (935,196, 1,088,732)	2667.8 (2449.5, 2922.6)	-0.4 (-2.4, 1.9)	1,507,392 (1,316,138, 1,727,380)	4763.1 (4097.9, 5444)	0 (-0.3, 0.3)	35,947 (20,992, 57,943)	88.5 (52.6, 139.1)	0 (-4.7, 5.3)
Algeria	1,111,122 (1,017,508, 1,224,563)	2667.2 (2448, 2920.7)	0 (-2.3, 2.2)	1,985,530 (1,702,553, 2,278,007)	4757.8 (4091.9, 5440.8)	0 (-0.1, 0)	37,639 (22,347, 58,301)	89.6 (53.1, 138.2)	0 (-4.5, 4.7)
Bahrain	37,030 (33,081, 41,824)	2628.4 (2404.6, 2865.2)	-0.2 (-2.5, 2.1)	74,479 (62,679, 88,087)	4717.6 (4057.9, 5391.7)	-0.2 (-0.4, 0)	1211 (736, 1834)	88.1 (52.9, 137.3)	-0.4 (-5.2, 4.5)
Egypt	2,143,848 (1,931,741, 2,384,042)	2206.9 (1984.8, 2454.9)	-1.5 (-4.4, 1.5)	4,465,049 (3,853,868, 5,117,502)	4741 (4080.1, 5424)	-0.3 (-0.4, -0.1)	68,217 (41,383, 106,456)	68.4 (41.7, 105.7)	-2 (-7.5, 3.7)
Iran (Islamic Republic of)	2,388,242 (2,163,553, 2,648,527)	2831.1 (2585.2, 3112.2)	0.3 (-1.3, 1.8)	4,595,841 (3,890,963, 5,342,153)	5237.3 (4459.6, 6025.5)	0.1 (-0.1, 0.3)	78,743 (47,930, 119,963)	94 (57.2, 145.2)	0.6 (-1.7, 2.8)
Iraq	1,110,564 (1,007,417, 1,212,301)	2663 (2430.3, 2918.6)	-0.1 (-2.1, 1.9)	1,881,432 (1,625,042, 2,158,367)	4760.6 (4092.3, 5441.9)	0 (0, 0)	38,401 (22,855, 60,208)	89.1 (54, 137.3)	0.3 (-4.4, 4.6)
Jordan	305,903 (281,116, 333,424)	2654 (2428.5, 2902)	-0.3 (-2.7, 1.9)	525,750 (454,646, 604,504)	4750.1 (4087.3, 5430)	-0.2 (-0.3, -0.1)	10,553 (6244, 16,505)	89.1 (53.6, 138.7)	-0.3 (-4.8, 4.2)
Kuwait	115,667 (104,284, 129,518)	2651.4 (2434.6, 2894)	0.8 (-1.4, 3.1)	223,152 (188,417, 263,227)	4739.9 (4078.1, 5420.8)	0.6 (0.2, 0.9)	3868 (2325, 6019)	89.2 (53.4, 138.4)	0.7 (-3.4, 5.2)
Lebanon	138,685 (126,603, 152,305)	2675.1 (2449.4, 2933.7)	0.1 (-2.1, 2.2)	251,925 (215,858, 289,305)	4779.9 (4110.5, 5463)	0.1 (0, 0.3)	4637 (2788, 7110)	89.8 (53.7, 137.5)	0.4 (-3.8, 5.7)
Libya	177,376 (161,449, 196,455)	2662.6 (2439.8, 2915.3)	0.4 (-1.7, 2.7)	333,709 (285,773, 387,314)	4755.8 (4091.5, 5437.8)	0.4 (0.2, 0.6)	5904 (3532, 9133)	89.2 (53.6, 139)	0.1 (-4.6, 4.7)
Morocco	954,966 (873,521, 1,047,916)	2668.3 (2440.2, 2917.2)	-0.1 (-2.3, 2)	1,732,826 (1,486,287, 1,993,426)	4761.3 (4095.4, 5445.1)	0.1 (0, 0.1)	32,033 (19,338, 49,673)	89.5 (54, 139)	-0.2 (-4.8, 4.9)
Oman	117,396 (105,908, 132,000)	2615.9 (2402, 2852.8)	-0.5 (-2.7, 1.5)	220,094 (186,343, 258,037)	4714.3 (4053.9, 5380.5)	-0.4 (-0.6, -0.3)	4004 (2419, 6276)	87.7 (52.9, 137)	-0.2 (-4.9, 4.7)
Palestine	131,061 (121,363, 141,947)	2667.4 (2443.2, 2916.2)	-0.3 (-2.4, 1.9)	212,759 (184,685, 243,471)	4762.2 (4097.3, 5445.9)	-0.2 (-0.4, -0.1)	4586 (2726, 7298)	89.2 (53.4, 139)	-0.4 (-4.9, 4.9)
Qatar	71,554 (63,478, 81,489)	2576.2 (2358.9, 2814.5)	-0.8 (-3.2, 1.5)	145,401 (121,725, 172,995)	4684.1 (4034.7, 5337.2)	-0.5 (-0.9, -0.2)	2386 (1425, 3655)	86.2 (52.1, 133)	-0.9 (-5.8, 4.3)
Saudi Arabia	923,945 (831,761, 1,033,982)	2635.9 (2410.4, 2883.3)	0 (-2.1, 2.3)	1,771,565 (1,499,985, 2,085,560)	4737.1 (4074.6, 5408.1)	0.2 (0.1, 0.3)	30,996 (18,903, 48,825)	88.3 (53, 137.9)	-0.1 (-4.7, 4.7)
Sudan	1,077,892 (992,738, 1,167,799)	2665.6 (2442, 2913.2)	-0.1 (-2.4, 2.1)	1,730,461 (1,502,767, 1,976,500)	4757.9 (4091.6, 5442.2)	0 (-0.1, 0.1)	37,893 (22,330, 60,491)	89.3 (53.4, 139.4)	-0.1 (-4.4, 4.5)
Syrian Arab Republic	384,407 (352,468, 420,630)	2679.5 (2466.9, 2933.4)	0.6 (-1.5, 2.9)	686,878 (588,603, 791,077)	4763.7 (4100, 5449.8)	0.2 (0, 0.5)	12,864 (7811, 20,035)	89.7 (54.4, 140)	0.2 (-4.6, 5)
Tunisia	309,517 (282,218, 342,747)	2672.1 (2454.4, 2921.4)	0.2 (-1.9, 2.4)	581,502 (495,333, 667,831)	4768.1 (4101.3, 5453.3)	0.2 (0.2, 0.3)	10,213 (6164, 15,470)	89.9 (53.9, 137.5)	0.1 (-4.5, 4.6)
Turkey	2,993,721 (2,748,618, 3,249,569)	3768.8 (3490.5, 4060.7)	0.1 (-2.9, 3.1)	4,235,198 (3,631,250, 4,870,849)	4867.3 (4203.1, 5558.1)	0 (-0.2, 0.2)	107,663 (63,418, 171,238)	138.7 (80.9, 222.6)	0.3 (-4.6, 5.3)
United Arab Emirates	230,844 (201,206, 267,656)	2594.1 (2369.6, 2834.1)	-0.3 (-2.6, 2.1)	485,868 (404,079, 593,641)	4679.9 (4021.9, 5342.3)	-0.5 (-0.8, -0.2)	7558 (4513, 11,811)	87 (52.1, 135.9)	-0.2 (-5.3, 4.6)
Yemen	835,090 (771,899, 907,163)	2670.1 (2448.5, 2929.2)	-0.1 (-2.2, 2.2)	1,313,588 (1,143,991, 1,495,412)	4762.2 (4096.5, 5446.4)	-0.2 (-0.4, -0.1)	29,363 (17,381, 46,119)	89 (53.8, 138.1)	0 (-4.5, 4.8)

### Individual country level

**Fig. 1:**
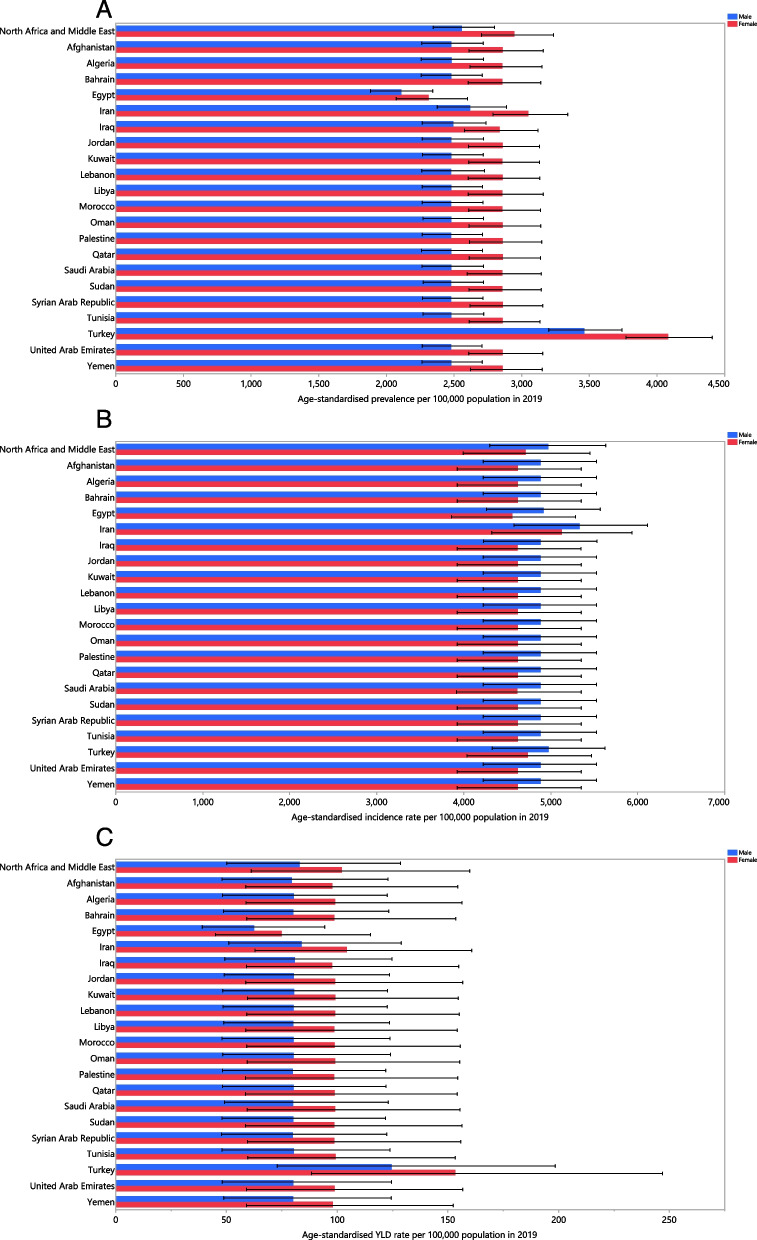
Age-standardised point prevalence (**A**), incidence rate (**B**), and YLD rate (**C**) for dermatitis (per 100,000 population) in the Middle East and North Africa region in 2019, by sex and country. YLD = year-lived with disability. (Generated from data available from http://ghdx.healthdata.org/gbd-results-tool)

In 2019, the age-standardised point prevalence of dermatitis varied from 2206.9 to 3768.8 per 100,000. Turkey [3768.8 (95% UI 3490.5 to 4060.7)], Iran [2831.1 (95% UI 2585.2 to 3112.2)], and the Syrian Arab Republic [2679.5 (95% UI 2466.9 to 2933.4)] exhibited the largest point prevalence, while Egypt [2206.9 (95% UI 1984.8 to 2454.9)], Qatar [2576.2 (95% UI 2358.9 to 2814.5)], and the United Arab Emirates [2594.1 (95% UI 2369.6 to 2834.1)] had the lowest (Table S2). Figure 1A displays the age-standardised point prevalence of dermatitis in 2019 by country and sex.

The age-standardised incidence rate of dermatitis ranged from 4679.9 to 5237.3 per 100,000. Iran [5237.3 (95% UI 4559.6 to 6025.5)], Turkey [4867.3 (95% UI 4203.1 to 5558.1)], and Lebanon [4779.9 (95% UI 4110.5 to 5463.0)] had the highest age-standardised incidence rates, while the United Arab Emirates [4679.9 (95% UI 4021.9 to 5342.3)], Qatar [4684.1 (95% UI 4034.7 to 5337.2)], and Oman [4714.3 (95% UI 4053.9 to 5380.5)] had the smallest (Table S3). The national age-standardised incidence rates of dermatitis in 2019 are shown by sex in Fig. 1B.

The age-standardised YLD rates of dermatitis in MENA ranged from 68.4 to 138.7 per 100,000. Turkey [138.7 (95% UI 80.9 to 222.6)], Iran [94.0 (95% UI 57.2 to 145.2)], and Tunisia [89.9 (95% UI 53.9 to 137.5)] had the largest age-standardised YLD rates in 2019, while Egypt [68.4 (95% UI 41.7 to 105.7)], Qatar [86.2 (95% UI 52.1 to 133.0)], and Oman [87.7 (95% UI 52.9 to 1370)] had the lowest (Table S4). The national age-standardised YLD rates of dermatitis in 2019 are shown by sex in Fig. 1C.

There were no significant increases in the age-standardised point prevalence of dermatitis in any of the MENA countries between 1990 and 2019 (Table S2 and Fig. S1). The biggest increases in the age-standardised incidence rates of dermatitis, from 1990 to 2019, were seen in Kuwait [0.6% (95% UI 0.2% to 0.9%)], Libya [0.4% (95% UI 0.2% to 0.6%)] and Saudi Arabia [0.2% (95% UI 0.1% to 0.3%)]. In contrast, Qatar [-0.5% (95% UI -0.9% to -0.2%)], the United Arab Emirates [-0.5% (95% UI -0.8% to -0.2%)], and Oman [-0.4% (95% UI -0.6% to -0.3%)] had the biggest decreases in the age-standardised incidence rates (Table S3 and Fig. S2). The age-standardised YLD rates of dermatitis did not change substantially in any of the MENA countries across the measurement period (Table S4 and Fig. S3).

### Age and sex patterns

**Fig. 2:**
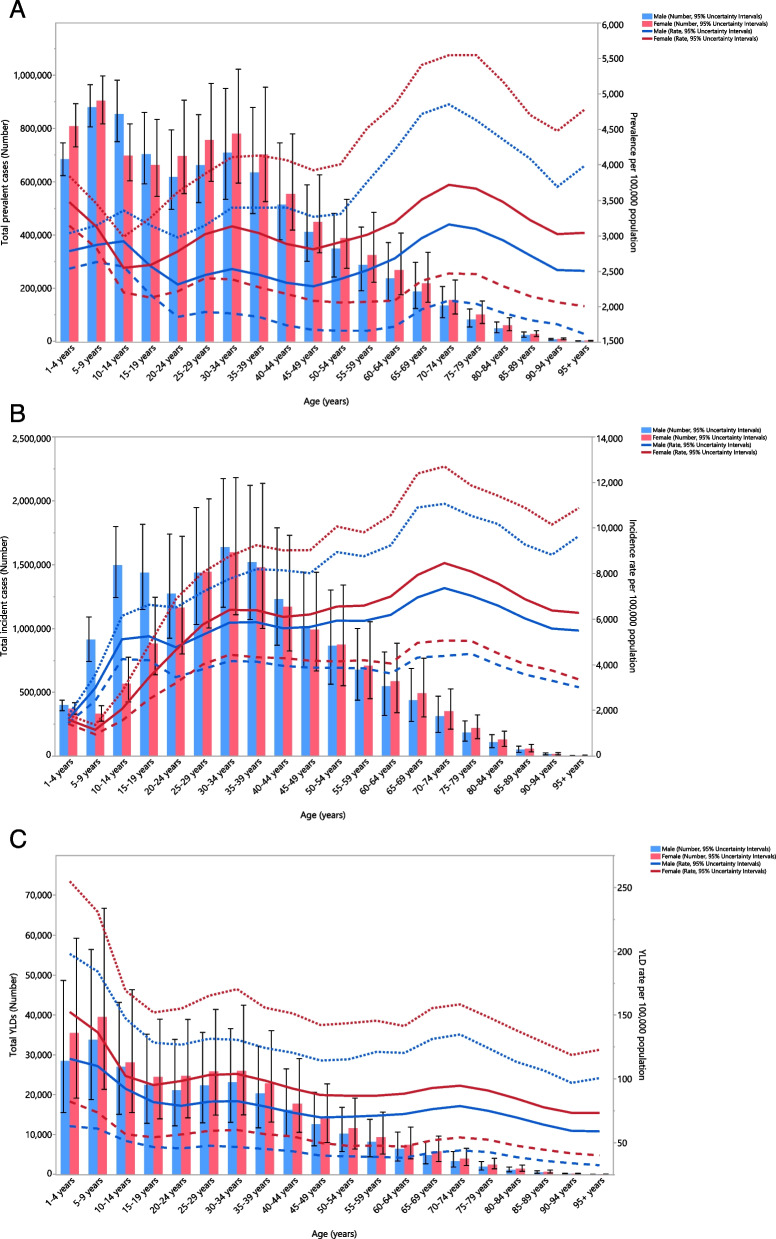
Number of prevalent cases and point prevalence (**A**), number of incident cases and incidence rate (**B**), and the number of YLDs and YLD rate (**C**) for dermatitis (per 100,000 population) in the Middle East and North Africa region, by age and sex in 2019; Dotted and dashed lines indicate 95% upper and lower uncertainty intervals, respectively. YLD = year-lived with disability. (Generated from data available from http://ghdx.healthdata.org/gbd-results-tool)

In 2019, the number of prevalent cases and the point prevalence of dermatitis were higher in women than men, but this was not statistically significant. The total number of prevalent cases of dermatitis were higher in the younger age groups, peaking in the 5 to 9 and 30 to 34 age groups, and then declined with advancing age. Unlike the overall counts, the point prevalence of dermatitis rose as age advanced, peaking in the 70 to 74 age bracket (Fig. 2A).

A higher number of incident cases of dermatitis were found among males up to 49 years old. The incidence rates of dermatitis were higher among males up to 20 to 24 age group, after which women had higher incidence rates, but these differences were not significant. Furthermore, the incident numbers were highest in the 30 to 34 age bracket, in both men and women, and then started to decline with advancing age. In contrast, the incidence rates showed an increasing trend throughout the age categories, reaching a peak among those aged 70 to 74 for both sexes (Fig. 2B).

Similar to the prevalence, women had a higher YLDs count and a higher YLD rate compared to men, but this difference was not significant. The YLD numbers and rates both peaked among those aged 1 to 4 and then decreased from the youngest age groups until they plateaued after the 50 to 54 age group (Fig. 2C).

**Fig. 3:**
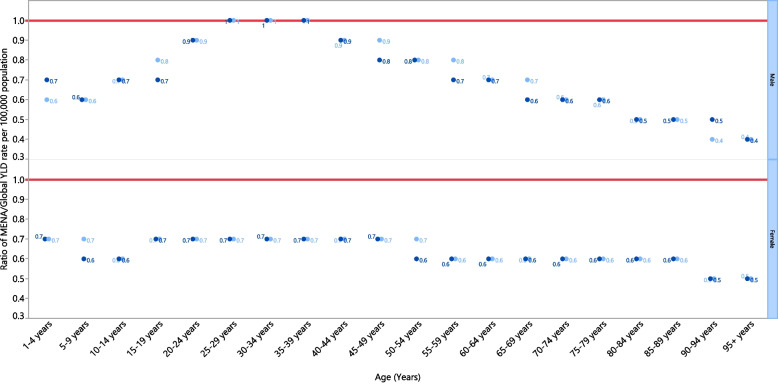
Ratio of the Middle East and North Africa region’s YLD rate to the global YLD rate of dermatitis by age group and sex, 1990–2019. YLD = year-lived with disability. (Generated from data available from http://ghdx.healthdata.org/gbd-results-tool)

The ratio between age-standardized YLD rates in MENA and the global rates for each sex and age category showed that females in MENA consistently maintained a lower YLD rate compared to the global rate in most age categories from 1990 to 2019. The male population had a similar YLD rate to the global rates in the 25 to 39 age categories, but were lower in every other age category (Fig. 3).

### The burden of dermatitis by type

**Fig. 4:**
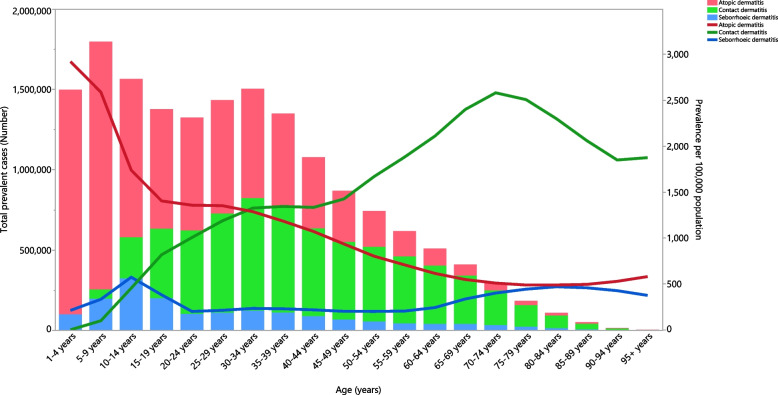
Number of prevalent cases and the age-standardised point prevalence of dermatitis (per 100,000 population) in the Middle East and North Africa region, by age and type in 2019. (Generated from data available from http://ghdx.healthdata.org/gbd-results-tool)

In 2019, the number of prevalent cases of dermatitis in MENA differed according to the type of dermatitis. In the earlier age groups (less than 19 years old) atopic dermatitis had the highest prevalence, while contact dermatitis was the most prevalent in the 25 to 39 age group and remained the most prevalent type of dermatitis in all older age groups. Seborrheic dermatitis had the lowest numbers, but its peak was seen in the 10 to 14 age group. The point prevalence of the types of dermatitis (per 100,000) followed different patterns, with atopic dermatitis being highest among those aged 1 to 4, while contact dermatitis reached its peak among those aged 70 to 74 (Fig. 4).

### Socio-demographic index (SDI)

**Fig. 5:**
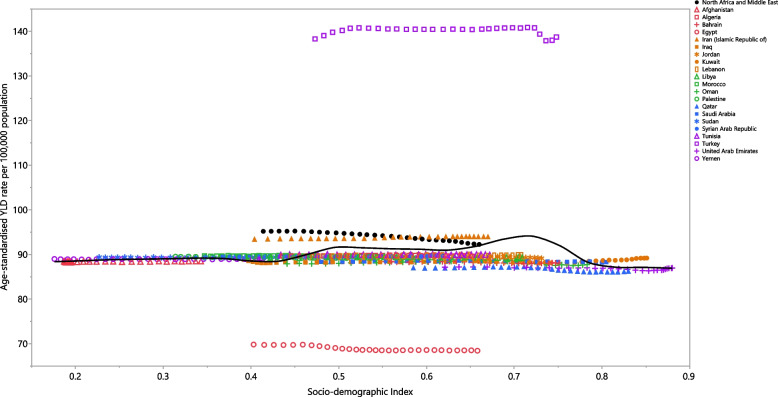
Age-standardised YLD rates of dermatitis for the 21 MENA countries in 2019, by SDI; Expected values based on the Socio-demographic Index and disease rates in all locations are shown as the black line. Each point shows the observed age-standardised YLD rate for each country in 2019. YLD = year-lived with disability. SDI = Socio-demographic Index (Generated from data available from http://ghdx.healthdata.org/gbd-results-tool)

In 2019, there wasn’t a clear relationship between the burden of dermatitis and SDI, although there is a slight peak in the burden of dermatitis at an SDI level of 0.7. Turkey and Iran exhibited higher-than-expected burdens, while Egypt and Qatar showed burdens that were lower than expected (Fig. 5).

## Discussion

There were small changes in the burden of dermatitis between 1990 and 2019. In all MENA countries, the age-standardised incidence rates of dermatitis were higher in males, while the age-standardised point prevalence and YLD rates were higher among females. However, it should be noted that the differences between sexes were not statistically significant. The point prevalence and incidence rate were higher among the elderly, while the YLD number and rate was higher among children. Moreover, no clear association was found between the regional burden of dermatitis and SDI.

Compared to the global burden of dermatitis, the MENA region exhibited a lower age-standardised incidence rate (4844.4 vs 5244.4 per 100,000), a lower age-standardised point prevalence (2744.6 vs 3653.4 per 100,000), and a lower age-standardised YLD rate (92.26 vs 131.67 per 100,000) [16]. This pattern was also evident in the MENA/Global YLD ratios, which were lower or equal to one for both sexes across all age groups in both 1990 and 2019. A major factor could be the under reporting and under diagnosis of dermatitis in the GBD study. Another factor that may have contributed to this is the absence of an analysis of risk factors and their individual burdens. Therefore, drawing conclusions about the factors behind these statistics is particularly challenging. Moreover, the largest age-standardised incidence rates were observed in Iran and Turkey, with only Egypt displaying a decline in the age-standardised point prevalence. The age-standardised YLD rate exhibited a comparable trend to the age-standardised point prevalence. We observed the age-standardised point prevalence of atopic dermatitis to be highest among individuals aged 1–4, whereas for seborrheic dermatitis, it was most prevalent in the 70–74 age category.

Previous research by Xue and colleagues found that atopic dermatitis accounted for a larger burden than contact dermatitis and seborrheic dermatitis [16]. This is in line with the hygiene hypothesis [19] and we hypothesize that higher socioeconomic development and industrialisation are some of the causes of this finding [20]. Moreover, the study’s limitations include underreporting cases of dermatitis in the region, with sparse data available on its incidence and prevalence, especially in low- and middle-income countries. The paucity of data should be taken into consideration when reading the results of the present research.

The higher age-standardised point prevalence and YLD rate in females, despite having a lower age-standardised incidence, can be explained by the higher severity of the disease and the more pronounced negative impact on women, possibly due to the higher importance of physical appearance among women, as has been suggested in the literature [21–23]. This result aligns with the findings of the earlier global study [20]. Females exhibited higher incidence rates across most age categories, while men displayed much higher incidence rates in the 1 to 24 age groups. In line with previous research, atopic dermatitis constituted the primary contributor to the dermatitis burden. Furthermore, atopic dermatitis is a predominantly male disease during childhood and predominantly female disease after adolescence [24, 25].

Overall, the age-standardised incidence of dermatitis increased with age. This is potentially due to changes in the disease severity with increasing age and the more subtle symptoms of the disease [26]. A higher age-standardised point prevalence of atopic dermatitis was observed earlier in life, which is somewhat in agreement with previous research [27, 28]. There was a peak in the age-standardised point prevalence of dermatitis in the 10–14 age group for seborrheic dermatitis. This coincides with the hormonal changes seen during puberty and the subsequent increase in sebaceous gland activity [29]. The highest prevalence of contact dermatitis was found among those who were aged 70–74. However, the largest number of cases were among those aged 30–34 years old, which coincides with occupational exposures. Among the elderly population, asteatosis dermatitis, triggered by skin dryness and aging, is the most common form of contact dermatitis [30]. In addition, contact dermatitis caused by nickel exposure from dental materials may be a contributing factor to the high prevalence of this condition in the elderly [31].

In contrast to the global research [16], we did not observe a clear relationship between SDI and the burden of dermatitis. This suggests that the epidemiology of dermatitis in the MENA region involves more diverse etiologies and risk factors. The disparity in findings can be attributed to the evaluation being conducted at the global level in the abovementioned article, whereas our study focused on the regional level.

Those with dermatitis commonly cite emotional distress, physical symptoms, sleep disturbances due to physical symptoms, and the burden of treatment as the key contributors to their lower quality of life [32]. The resultant excessive daytime tiredness could also cause impairment in activities [33] and it is the degree of impairment that has been found to be the most important predictor of a low quality of life [34]. Dermatitis has been strongly correlated with urbanization and associated lifestyle [19, 35]. However, the most important risk factors proposed for atopic dermatitis are a family history of atopic disease, maternal smoking, and active smoking [36]. Emerging research has placed the focus on prenatal risk factors [37], many of which can be modified by better screening, prenatal care, and education. Smoking remains an important public health issue in the MENA region, particularly when compared to the rest of the world [38].

A recent meta-analysis found that exposure to wet work was associated with contact dermatitis [39]. Moreover, irritant contact dermatitis has also been linked to other factors, including exposure to different chemicals, soluble oils, detergents, occlusion by gloves, contact with plants, and mechanical friction [40]. In workplace environments, it is crucial to consider contact dermatitis due to personal protective equipment, for example in healthcare workers who wear facial masks for prolonged periods [41]. Nickel is the most common contact allergen worldwide [42, 43]. While exposure to nickel in occupation settings has decreased over the years, it is still widely used in numerous household items, surgical implants, and dental materials [31]. The 1994 EU Nickel Directive led to a substantial reduction in Nickel content across household and industrial items, as many companies adhered to these regulations. Consequently, the prevalence of this allergy decreased [44]. However, numerous countries have not universally implemented similar measures for domestically produced and consumed goods. This has resulted in Nickel-induced contact dermatitis persisting as the most prevalent form, as evidenced by recent studies in Turkey and Iran [45, 46]. Therefore, maintaining vigilance regarding potential Nickel exposure remains important.

Patients with contact dermatitis might encounter challenges in selecting and maintaining certain professions, since their condition limits their ability to work in environments where they might be exposed to the aforementioned irritants [47]. Furthermore, studies have shown that ongoing exposure to these irritants is predictive of a lower likelihood of complete healing [40]. Hence, implementing comprehensive workplace safety regulations could substantially improve the current burden of this disease. Several of these interventions have been shown to help individuals retain their jobs [48].

Emotional distress and social isolation are the most significant factors affecting the quality of life among individuals with seborrheic dermatitis, particularly those with scalp lesions and shedding of scales [49]. Women, younger individual, and more educated people seem to experience greater distress from this condition [22]. The most important risk factors for seborrheic dermatitis are male gender, Malassezia yeast, host epidermal conditions, sebaceous secretion, and the status of the immune response [6, 29]. The typical pathogenesis of the disease is explained by an initial higher production of lipids by the sebaceous glands, followed by colonization by Malassezia, leading to inflammation and a host reaction [50]. In terms of environmental and lifestyle risk factors, zinc deficiency, and high humidity have been identified as important factors in the susceptibility to seborrheic dermatitis [51]. Similar to other diseases with a microbial source, antibiotic resistance is a serious public health concern [52, 53]. Therefore, it is essential to establish better guidelines and programs to promote the responsible prescription of antifungal agents to help mitigate this problem. A warm and humid climate has been linked to a lower occurrence of atopic dermatitis [54]. Conversely, hot weather has been associated with lower quality of life and consequently a higher burden in seborrheic dermatitis [55]. However, it is important to note that atopic dermatitis is a substantial contributor to the overall burden of dermatitis. This partially explains the lower dermatitis attributable YLDs in MENA, when compared to the worldwide average.

## Limitations

The most important limitation of the present research is the quality of the data available in the MENA region. Similar to other GBD studies on this subject, the statistics presented here are based upon a limited number of epidemiology studies of dermatitis in the region. In addition, insurance claims data and registry data from other countries were also used, which might not exactly represent the population of the MENA region [12]. Another limitation is the potential underreporting of dermatitis, particularly atopic dermatitis, which can have very few symptoms, especially in the elderly. Finally, combining and analysing the three different types of dermatitis might create biases in the interpretation of the results, since each type has a different trend that might mask the trends of the other types of this disease.

## Conclusions

The dermatitis burden in MENA remained relatively stable from 1990 to 2019. Future prevention efforts should be focused on improving access to appropriate healthcare services, as well as health education and prenatal education. We suggest that future studies focus on quantifying personal occupational exposure in workplaces where individuals face regular exposure to irritants associated with dermatitis. Implementing control measures based on these quantitative studies and enhancing workplace safety regulations should also help to reduce occupational exposure to these irritants.

### Supplementary Information

**Additional file 1: Table S1.** Health states for dermatitis and the associated disability weights from the Global Burden of Disease 2019 Study.

**Additional file 2: Table S2.** Prevalence of dermatitis in 1990 and 2019 and the percentage change in the age-standardised rates (ASRs) per 100,000 in the Middle East and North Africa region (Generated from data available from http://ghdx.healthdata.org/gbd-results-tool).

**Additional file 3: Table S3.** Incidence of dermatitis in 1990 and 2019 and the percentage change in the age-standardised rates (ASRs) per 100,000 in the Middle East and North Africa region (Generated from data available from http://ghdx.healthdata.org/gbd-results-tool).

**Additional file 4: Table S4.** YLDs due to dermatitis in 1990 and 2019 and the percentage change in the age-standardised rates (ASRs) per 100,000 in the Middle East and North Africa region (Generated from data available from http://ghdx.healthdata.org/gbd-results-tool).

**Additional file 5: Figure S1.** The percentage change in the age-standardised point prevalence of dermatitis in the Middle East and North Africa region from 1990 to 2019, by sex and country. (Generated from data available from http://ghdx.healthdata.org/gbd-results-tool).

**Additional file 6: Figure S2.** The percentage change in the age-standardised incidence rate of dermatitis in the Middle East and North Africa region from 1990 to 2019, by sex and country. (Generated from data available from http://ghdx.healthdata.org/gbd-results-tool).

**Additional file 7: Figure S3.** The percentage change in the age-standardised YLD rate of dermatitis in the Middle East and North Africa region from 1990 to 2019, by sex and country. YLD= year-lived with disability. (Generated from data available from http://ghdx.healthdata.org/gbd-results-tool).

## Data Availability

The data used for these analyses are all publicly available at http://ghdx.healthdata.org/gbd-results-tool.
