# Supplementary material for: The burden of dermatitis from 1990–2019 in the Middle East and North Africa region
Source: BMC Public Health. 2024 Feb 7;24:399. doi: 10.1186/s12889-024-17836-z (PMC10848450; doi:10.1186/s12889-024-17836-z)
Supplement: Supplementary file 1 — Additional file 1: Table S1. Health states for dermatitis and the associated disability weights from the Global Burden of Disease 2019 Study. [file 12889_2024_17836_MOESM1_ESM.docx]

**Table S1:** Health states for dermatitis and the associated disability weights from the Global Burden of Disease 2019 Study.

| Sequela | Severity level | Lay description | Disability weight (95% confidence interval) |
| --- | --- | --- | --- |
| Mild atopic dermatitis | Disfigurement level 1, with itch/pain | The person has a slight visible physical deformity that is sometimes sore or itchy. Others notice the deformity, which causes some worry and discomfort. | 0.027 (0.015–0.042) |
| Moderate atopic dermatitis | Disfigurement level 2, with itch/pain | The person has a visible physical deformity that is sore and itchy. Other people stare and comment, which causes the person to worry. The person has trouble sleeping and concentrating. | 0.188 (0.124–0.267) |
| Severe atopic dermatitis | Disfigurement level 3, with itch/pain | The person has an obvious physical deformity that is very painful and itchy. The physical deformity makes others uncomfortable, which causes the person to avoid social contact, feel worried, sleep poorly, and think about suicide. | 0.576 (0.401–0.731) |
| Mild contact dermatitis | Disfigurement level 1, with itch/pain | The person has a slight, visible physical deformity that is sometimes sore or itchy. Others notice the deformity, which causes some worry and discomfort. | 0.027 (0.015–0.042) |
| Moderate contact dermatitis | Disfigurement level 2, with itch/pain | The person has a visible physical deformity that is sore and itchy. Other people stare and comment, which causes the person to worry. The person has trouble sleeping and concentrating. | 0.188 (0.124–0.267) |
| Symptomatic seborrhoeic dermatitis | Disfigurement level 1, with itch/pain | The person has a slight visible physical deformity that is sometimes sore or itchy. Others notice the deformity, which causes some worry and discomfort. | 0.027 (0.015–0.042) |
