# Supplementary material for: The burden of dermatitis from 1990–2019 in the Middle East and North Africa region
Source: BMC Public Health. 2024 Feb 7;24:399. doi: 10.1186/s12889-024-17836-z (PMC10848450; doi:10.1186/s12889-024-17836-z)
Supplement: Supplementary file 2 — Additional file 2: Table S2. Prevalence of dermatitis in 1990 and 2019 and the percentage change in the age-standardised rates (ASRs) per 100,000 in the Middle East and North Africa region (Generated from data available from http://ghdx.healthdata.org/gbd-results-tool). [file 12889_2024_17836_MOESM2_ESM.docx]

| **Table S2: Prevalence of dermatitis in 1990 and 2019 and the percentage change in the age-standardised rates (ASRs) per 100,000 in the Middle East and North Africa region**  **(Generated from data available from http://ghdx.healthdata.org/gbd-results-tool)** | | | | | |
| --- | --- | --- | --- | --- | --- |
|  | **1990** | | **2019** | | **Percentage change in ASRs per 100,000 (95% UI)** |
|  | **No (95% UI)** | **ASRs per 100,000 (95% UI)** | **No (95% UI)** | **ASRs per 100,000 (95% UI)** |  |
| **North Africa and Middle East** | **9639046 (8987610 , 10385385)** | **2809.6 (2584 , 3067.2)** | **16581955 (15229446 , 18129071)** | **2744.6 (2517.8 , 3003.1)** | **-2.3 (-3.2 , -1.5)** |
| **Afghanistan** | **301015 (278167 , 324401)** | **2677.5 (2447.6 , 2926)** | **1006275 (935196 , 1088732)** | **2667.8 (2449.5 , 2922.6)** | **-0.4 (-2.4 , 1.9)** |
| **Algeria** | **671791 (623153 , 723964)** | **2667.1 (2452.1 , 2911.3)** | **1111122 (1017508 , 1224563)** | **2667.2 (2448 , 2920.7)** | **0 (-2.3 , 2.2)** |
| **Bahrain** | **13200 (12048 , 14578)** | **2634.2 (2410.3 , 2883.9)** | **37030 (33081 , 41824)** | **2628.4 (2404.6 , 2865.2)** | **-0.2 (-2.5 , 2.1)** |
| **Egypt** | **1206898 (1099155 , 1323237)** | **2239.8 (2016.2 , 2484.9)** | **2143848 (1931741 , 2384042)** | **2206.9 (1984.8 , 2454.9)** | **-1.5 (-4.4 , 1.5)** |
| **Iran (Islamic Republic of)** | **1617592 (1505497 , 1751297)** | **2822.8 (2582 , 3108.7)** | **2388242 (2163553 , 2648527)** | **2831.1 (2585.2 , 3112.2)** | **0.3 (-1.3 , 1.8)** |
| **Iraq** | **465144 (427942 , 505800)** | **2664.9 (2433.5 , 2924.3)** | **1110564 (1007417 , 1212301)** | **2663 (2430.3 , 2918.6)** | **-0.1 (-2.1 , 1.9)** |
| **Jordan** | **99170 (91964 , 107325)** | **2661.7 (2434.1 , 2904.4)** | **305903 (281116 , 333424)** | **2654 (2428.5 , 2902)** | **-0.3 (-2.7 , 1.9)** |
| **Kuwait** | **45797 (41689 , 50270)** | **2629.6 (2414.7 , 2868.2)** | **115667 (104284 , 129518)** | **2651.4 (2434.6 , 2894)** | **0.8 (-1.4 , 3.1)** |
| **Lebanon** | **86786 (80425 , 93951)** | **2673.1 (2452.7 , 2923.2)** | **138685 (126603 , 152305)** | **2675.1 (2449.4 , 2933.7)** | **0.1 (-2.1 , 2.2)** |
| **Libya** | **111623 (103764 , 120238)** | **2653.3 (2435.2 , 2899.7)** | **177376 (161449 , 196455)** | **2662.6 (2439.8 , 2915.3)** | **0.4 (-1.7 , 2.7)** |
| **Morocco** | **670172 (618652 , 725945)** | **2672 (2459.1 , 2914.6)** | **954966 (873521 , 1047916)** | **2668.3 (2440.2 , 2917.2)** | **-0.1 (-2.3 , 2)** |
| **Oman** | **50718 (46761 , 54861)** | **2628.9 (2415.4 , 2867.7)** | **117396 (105908 , 132000)** | **2615.9 (2402 , 2852.8)** | **-0.5 (-2.7 , 1.5)** |
| **Palestine** | **54650 (50631 , 58950)** | **2675.7 (2453.2 , 2927.8)** | **131061 (121363 , 141947)** | **2667.4 (2443.2 , 2916.2)** | **-0.3 (-2.4 , 1.9)** |
| **Qatar** | **11304 (10218 , 12598)** | **2597.4 (2375.6 , 2838.9)** | **71554 (63478 , 81489)** | **2576.2 (2358.9 , 2814.5)** | **-0.8 (-3.2 , 1.5)** |
| **Saudi Arabia** | **419428 (388877 , 452689)** | **2634.6 (2417.2 , 2869)** | **923945 (831761 , 1033982)** | **2635.9 (2410.4 , 2883.3)** | **0 (-2.1 , 2.3)** |
| **Sudan** | **530410 (493089 , 572246)** | **2669.6 (2453.1 , 2912.9)** | **1077892 (992738 , 1167799)** | **2665.6 (2442 , 2913.2)** | **-0.1 (-2.4 , 2.1)** |
| **Syrian Arab Republic** | **340518 (316048 , 366193)** | **2664 (2441.1 , 2911.5)** | **384407 (352468 , 420630)** | **2679.5 (2466.9 , 2933.4)** | **0.6 (-1.5 , 2.9)** |
| **Tunisia** | **223969 (206504 , 242342)** | **2667.2 (2440.2 , 2908.9)** | **309517 (282218 , 342747)** | **2672.1 (2454.4 , 2921.4)** | **0.2 (-1.9 , 2.4)** |
| **Turkey** | **2301244 (2142473 , 2471205)** | **3764.8 (3498.5 , 4060.2)** | **2993721 (2748618 , 3249569)** | **3768.8 (3490.5 , 4060.7)** | **0.1 (-2.9 , 3.1)** |
| **United Arab Emirates** | **47892 (43495 , 53101)** | **2601.2 (2376.6 , 2829.6)** | **230844 (201206 , 267656)** | **2594.1 (2369.6 , 2834.1)** | **-0.3 (-2.6 , 2.1)** |
| **Yemen** | **363243 (337746 , 389524)** | **2672.4 (2454.3 , 2920.9)** | **835090 (771899 , 907163)** | **2670.1 (2448.5 , 2929.2)** | **-0.1 (-2.2 , 2.2)** |
