# Supplementary material for: The burden of dermatitis from 1990–2019 in the Middle East and North Africa region
Source: BMC Public Health. 2024 Feb 7;24:399. doi: 10.1186/s12889-024-17836-z (PMC10848450; doi:10.1186/s12889-024-17836-z)
Supplement: Supplementary file 3 — Additional file 3: Table S3. Incidence of dermatitis in 1990 and 2019 and the percentage change in the age-standardised rates (ASRs) per 100,000 in the Middle East and North Africa region (Generated from data available from http://ghdx.healthdata.org/gbd-results-tool). [file 12889_2024_17836_MOESM3_ESM.docx]

| **Table S3: Incidence of dermatitis in 1990 and 2019 and the percentage change in the age-standardised rates (ASRs) per 100,000 in the Middle East and North Africa region**  **(Generated from data available from http://ghdx.healthdata.org/gbd-results-tool)** | | | | | |
| --- | --- | --- | --- | --- | --- |
|  | **1990** | | **2019** | | **Percentage change in ASRs per 100,000 (95% UI)** |
|  | **No (95% UI)** | **ASRs per 100,000 (95% UI)** | **No (95% UI)** | **ASRs per 100,000 (95% UI)** |  |
| **North Africa and Middle East** | **14488599 (12607667 , 16455768)** | **4846.5 (4160.9 , 5545.4)** | **28989852 (24931092 , 33310421)** | **4844.4 (4160.3 , 5539.7)** | **0 (-0.2 , 0.1)** |
| **Afghanistan** | **460875 (402039 , 522944)** | **4762.8 (4097.1 , 5450.8)** | **1507392 (1316138 , 1727380)** | **4763.1 (4097.9 , 5444)** | **0 (-0.3 , 0.3)** |
| **Algeria** | **1039453 (909168 , 1180114)** | **4759.2 (4092.7 , 5442.9)** | **1985530 (1702553 , 2278007)** | **4757.8 (4091.9 , 5440.8)** | **0 (-0.1 , 0)** |
| **Bahrain** | **22545 (19223 , 26042)** | **4727.4 (4068.1 , 5402.1)** | **74479 (62679 , 88087)** | **4717.6 (4057.9 , 5391.7)** | **-0.2 (-0.4 , 0)** |
| **Egypt** | **2336687 (2031278 , 2656054)** | **4753.3 (4091.6 , 5436.1)** | **4465049 (3853868 , 5117502)** | **4741 (4080.1 , 5424)** | **-0.3 (-0.4 , -0.1)** |
| **Iran (Islamic Republic of)** | **2532626 (2198511 , 2891329)** | **5231.9 (4454.8 , 6022.6)** | **4595841 (3890963 , 5342153)** | **5237.3 (4459.6 , 6025.5)** | **0.1 (-0.1 , 0.3)** |
| **Iraq** | **695609 (609521 , 786755)** | **4759.9 (4091.2 , 5441.8)** | **1881432 (1625042 , 2158367)** | **4760.6 (4092.3 , 5441.9)** | **0 (0 , 0)** |
| **Jordan** | **149902 (131537 , 171230)** | **4758.4 (4093.2 , 5438.9)** | **525750 (454646 , 604504)** | **4750.1 (4087.3 , 5430)** | **-0.2 (-0.3 , -0.1)** |
| **Kuwait** | **78051 (66578 , 90280)** | **4712.9 (4056.8 , 5386.8)** | **223152 (188417 , 263227)** | **4739.9 (4078.1 , 5420.8)** | **0.6 (0.2 , 0.9)** |
| **Lebanon** | **141625 (123211 , 161422)** | **4774.2 (4105.3 , 5458.1)** | **251925 (215858 , 289305)** | **4779.9 (4110.5 , 5463)** | **0.1 (0 , 0.3)** |
| **Libya** | **169212 (148288 , 191542)** | **4736.7 (4072.3 , 5417)** | **333709 (285773 , 387314)** | **4755.8 (4091.5 , 5437.8)** | **0.4 (0.2 , 0.6)** |
| **Morocco** | **1073467 (931707 , 1223857)** | **4756.6 (4091.4 , 5442.2)** | **1732826 (1486287 , 1993426)** | **4761.3 (4095.4 , 5445.1)** | **0.1 (0 , 0.1)** |
| **Oman** | **79164 (68577 , 90422)** | **4734.1 (4074.7 , 5403.5)** | **220094 (186343 , 258037)** | **4714.3 (4053.9 , 5380.5)** | **-0.4 (-0.6 , -0.3)** |
| **Palestine** | **79658 (70131 , 90275)** | **4773 (4104.9 , 5458.5)** | **212759 (184685 , 243471)** | **4762.2 (4097.3 , 5445.9)** | **-0.2 (-0.4 , -0.1)** |
| **Qatar** | **20367 (17324 , 23884)** | **4707.3 (4045.3 , 5366.5)** | **145401 (121725 , 172995)** | **4684.1 (4034.7 , 5337.2)** | **-0.5 (-0.9 , -0.2)** |
| **Saudi Arabia** | **654355 (566824 , 746239)** | **4727.4 (4063.8 , 5396.4)** | **1771565 (1499985 , 2085560)** | **4737.1 (4074.6 , 5408.1)** | **0.2 (0.1 , 0.3)** |
| **Sudan** | **803422 (702977 , 910614)** | **4757.3 (4091.8 , 5443.8)** | **1730461 (1502767 , 1976500)** | **4757.9 (4091.6 , 5442.2)** | **0 (-0.1 , 0.1)** |
| **Syrian Arab Republic** | **503039 (442410 , 569779)** | **4754.3 (4089.4 , 5437.9)** | **686878 (588603 , 791077)** | **4763.7 (4100 , 5449.8)** | **0.2 (0 , 0.5)** |
| **Tunisia** | **362908 (315237 , 412462)** | **4757.5 (4092 , 5442.5)** | **581502 (495333 , 667831)** | **4768.1 (4101.3 , 5453.3)** | **0.2 (0.2 , 0.3)** |
| **Turkey** | **2679014 (2337664 , 3043314)** | **4866.4 (4196.8 , 5554.3)** | **4235198 (3631250 , 4870849)** | **4867.3 (4203.1 , 5558.1)** | **0 (-0.2 , 0.2)** |
| **United Arab Emirates** | **82940 (70526 , 96891)** | **4703.8 (4039.8 , 5369.8)** | **485868 (404079 , 593641)** | **4679.9 (4021.9 , 5342.3)** | **-0.5 (-0.8 , -0.2)** |
| **Yemen** | **513935 (451569 , 578806)** | **4773.3 (4107.1 , 5456.3)** | **1313588 (1143991 , 1495412)** | **4762.2 (4096.5 , 5446.4)** | **-0.2 (-0.4 , -0.1)** |
