# Supplementary material for: The burden of dermatitis from 1990–2019 in the Middle East and North Africa region
Source: BMC Public Health. 2024 Feb 7;24:399. doi: 10.1186/s12889-024-17836-z (PMC10848450; doi:10.1186/s12889-024-17836-z)
Supplement: Supplementary file 4 — Additional file 4: Table S4. YLDs due to dermatitis in 1990 and 2019 and the percentage change in the age-standardised rates (ASRs) per 100,000 in the Middle East and North Africa region (Generated from data available from http://ghdx.healthdata.org/gbd-results-tool). [file 12889_2024_17836_MOESM4_ESM.docx]

| **Table S4: YLDs due to dermatitis in 1990 and 2019 and the percentage change in the age-standardised rates (ASRs) per 100,000 in the Middle East and North Africa region**  **(Generated from data available from http://ghdx.healthdata.org/gbd-results-tool)** | | | | | |
| --- | --- | --- | --- | --- | --- |
|  | **1990** | | **2019** | | **Percentage change in ASRs per 100,000 (95% UI)** |
|  | **No (95% UI)** | **ASRs per 100,000 (95% UI)** | **No (95% UI)** | **ASRs per 100,000 (95% UI)** |  |
| **North Africa and Middle East** | **344359 (203281 , 547180)** | **95.2 (57.2 , 148.5)** | **565253 (339590 , 883269)** | **92.3 (55.6 , 143.4)** | **-3.1 (-4.6 , -1.6)** |
| **Afghanistan** | **10497 (6175 , 16534)** | **88.4 (52.5 , 135.8)** | **35947 (20992 , 57943)** | **88.5 (52.6 , 139.1)** | **0 (-4.7 , 5.3)** |
| **Algeria** | **23875 (13889 , 37541)** | **89.7 (53.7 , 140.4)** | **37639 (22347 , 58301)** | **89.6 (53.1 , 138.2)** | **0 (-4.5 , 4.7)** |
| **Bahrain** | **462 (274 , 722)** | **88.4 (53 , 138.2)** | **1211 (736 , 1834)** | **88.1 (52.9 , 137.3)** | **-0.4 (-5.2 , 4.5)** |
| **Egypt** | **39496 (23722 , 61521)** | **69.8 (42.2 , 106.6)** | **68217 (41383 , 106456)** | **68.4 (41.7 , 105.7)** | **-2 (-7.5 , 3.7)** |
| **Iran (Islamic Republic of)** | **57387 (34013 , 90641)** | **93.4 (56.5 , 144)** | **78743 (47930 , 119963)** | **94 (57.2 , 145.2)** | **0.6 (-1.7 , 2.8)** |
| **Iraq** | **16597 (9710 , 26785)** | **88.8 (53.6 , 138.5)** | **38401 (22855 , 60208)** | **89.1 (54 , 137.3)** | **0.3 (-4.4 , 4.6)** |
| **Jordan** | **3563 (2100 , 5638)** | **89.4 (54 , 139.1)** | **10553 (6244 , 16505)** | **89.1 (53.6 , 138.7)** | **-0.3 (-4.8 , 4.2)** |
| **Kuwait** | **1606 (947 , 2529)** | **88.6 (53.1 , 136.7)** | **3868 (2325 , 6019)** | **89.2 (53.4 , 138.4)** | **0.7 (-3.4 , 5.2)** |
| **Lebanon** | **3009 (1794 , 4677)** | **89.4 (53.8 , 139.3)** | **4637 (2788 , 7110)** | **89.8 (53.7 , 137.5)** | **0.4 (-3.8 , 5.7)** |
| **Libya** | **3992 (2350 , 6378)** | **89.1 (53.4 , 138.3)** | **5904 (3532 , 9133)** | **89.2 (53.6 , 139)** | **0.1 (-4.6 , 4.7)** |
| **Morocco** | **23539 (13822 , 37274)** | **89.7 (53.6 , 139.9)** | **32033 (19338 , 49673)** | **89.5 (54 , 139)** | **-0.2 (-4.8 , 4.9)** |
| **Oman** | **1808 (1084 , 2892)** | **87.9 (53.6 , 137.1)** | **4004 (2419 , 6276)** | **87.7 (52.9 , 137)** | **-0.2 (-4.9 , 4.7)** |
| **Palestine** | **1969 (1147 , 3163)** | **89.5 (53.7 , 139.2)** | **4586 (2726 , 7298)** | **89.2 (53.4 , 139)** | **-0.4 (-4.9 , 4.9)** |
| **Qatar** | **392 (236 , 613)** | **87 (52.4 , 135.2)** | **2386 (1425 , 3655)** | **86.2 (52.1 , 133)** | **-0.9 (-5.8 , 4.3)** |
| **Saudi Arabia** | **14973 (8934 , 23717)** | **88.3 (53.8 , 136.7)** | **30996 (18903 , 48825)** | **88.3 (53 , 137.9)** | **-0.1 (-4.7 , 4.7)** |
| **Sudan** | **18884 (11095 , 29987)** | **89.4 (53.7 , 139.8)** | **37893 (22330 , 60491)** | **89.3 (53.4 , 139.4)** | **-0.1 (-4.4 , 4.5)** |
| **Syrian Arab Republic** | **12266 (7238 , 19497)** | **89.5 (54.2 , 138)** | **12864 (7811 , 20035)** | **89.7 (54.4 , 140)** | **0.2 (-4.6 , 5)** |
| **Tunisia** | **7853 (4685 , 12494)** | **89.8 (54.3 , 141.3)** | **10213 (6164 , 15470)** | **89.9 (53.9 , 137.5)** | **0.1 (-4.5 , 4.6)** |
| **Turkey** | **87151 (49671 , 139857)** | **138.3 (79.9 , 221.3)** | **107663 (63418 , 171238)** | **138.7 (80.9 , 222.6)** | **0.3 (-4.6 , 5.3)** |
| **United Arab Emirates** | **1680 (995 , 2664)** | **87.1 (51.9 , 135.1)** | **7558 (4513 , 11811)** | **87 (52.1 , 135.9)** | **-0.2 (-5.3 , 4.6)** |
| **Yemen** | **13129 (7690 , 20969)** | **89 (53.6 , 136.7)** | **29363 (17381 , 46119)** | **89 (53.8 , 138.1)** | **0 (-4.5 , 4.8)** |
