# Supplementary material for: The burden of dermatitis from 1990–2019 in the Middle East and North Africa region
Source: BMC Public Health. 2024 Feb 7;24:399. doi: 10.1186/s12889-024-17836-z (PMC10848450; doi:10.1186/s12889-024-17836-z)
Supplement: Supplementary file 6 — Additional file 6: Figure S2. The percentage change in the age-standardised incidence rate of dermatitis in the Middle East and North Africa region from 1990 to 2019, by sex and country. (Generated from data available from http://ghdx.healthdata.org/gbd-results-tool). [file 12889_2024_17836_MOESM6_ESM.docx]

**
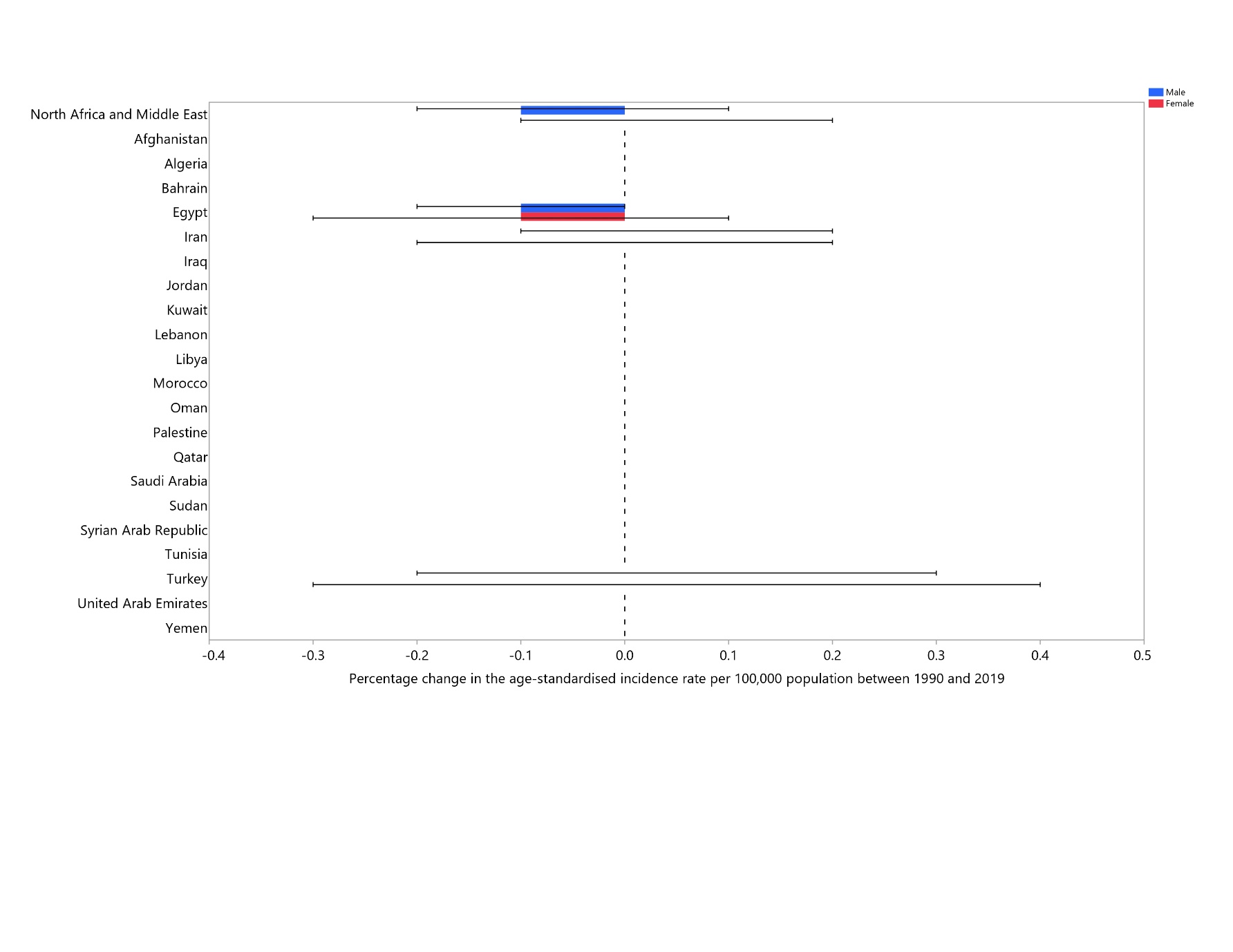
**

**Figure S2:** The percentage change in the age-standardised incidence rate of dermatitis in the Middle East and North Africa region from 1990 to 2019, by sex and country. (Generated from data available from <http://ghdx.healthdata.org/gbd-results-tool>).
